# Profiling Police Forces against Stress: Risk and Protective Factors for Post-Traumatic Stress Disorder and Burnout in Police Officers

**DOI:** 10.3390/ijerph19159218

**Published:** 2022-07-28

**Authors:** Royce Anders, Lauriane Willemin-Petignat, Cornelia Rolli Salathé, Andrea C. Samson, Benjamin Putois

**Affiliations:** 1 EPSYLON Laboratory, Department of Psychology, University Paul Valéry Montpellier 3, F34000 Montpellier, France; 2 EMC Laboratory, Institute of Psychology, University of Lyon 2, F69500 Bron, France; 3 Faculty of Psychology, Unidistance Suisse, 3900 Brig, Switzerland; cornelia.rolli@fernuni.ch (C.R.S.); andrea.samson@unidistance.ch (A.C.S.); benjamin.putois@unidistance.ch (B.P.); 4 Occupational Health Psychology, Faculty of Psychology, University of Fribourg, 1700 Fribourg, Switzerland; 5 Institute of Special Education, University of Fribourg, 1700 Fribourg, Switzerland; 6 Lyon Neuroscience Research Center, INSERM, CNRS, University of Lyon 1, F69675 Bron, France

**Keywords:** police, trauma, PTSD, burnout, stress, coping strategies, personality

## Abstract

Police officers are frequently exposed to highly stressful situations at work and have an increased risk to develop symptoms of post-traumatic stress disorder (PTSD) and burnout (BO). It is currently not well understood which officers are most at risk to develop these disorders. The aim of this study was to determine which coping strategies and personality traits could act as protective or risk factors in relation to PTSD and BO. The second aim, in the interest of designating preventive and therapeutical measures, was to determine whether certain profiles of police officers could be identified as high risk for developing mental disorders. Herein, 1073 French-speaking police officers in Switzerland reported in an online survey about their PTSD and BO symptoms, anxiety, depression, suicide ideation, coping strategies, occupational stress, and personality factors. The cluster analysis highlighted three principal profiles of police officers: those who are not at risk of developing pathologies because they are not exposed or insensitive to these stressors, and those who are, among which personality and coping strategies oriented the risk of developing PTSD or BO. These same protective and risk factors were also corroborated in the linear and logistic regression analyses. These results may suggest that a crucial opportunity for mitigating mental health issues in the force could consist of screening recruits for risk-related personality traits and orienting them towards psychological training programs for the development of functional coping strategies.

## 1. Introduction

Careers in policing are well known to involve repeated exposure to significantly stressful events and environments [1,2,3]. Police occupational stress, whether it is operational: i.e., linked to the unit and role of the officer (armed threats, motor accidents, civil aggression [4], death, child abuse) or organizational [5] i.e., linked to the management and logistics of the officer’s role (nonstandard working hours, understaffing, work relation abuse, lack of job satisfaction), is known to be maintained chronically throughout a police career. As a result, police careers have been markedly associated with consequential health risks [6,7,8]. Among these, mental disorders especially, such as posttraumatic stress disorder (PTSD), burnout (BO) and suicide ideation (SI) have been linked to police occupational stress [9,10,11,12].

PTSD corresponds to a severe anxiety disorder lasting for numerous years, which is first developed generally one month after having been confronted with a triggering traumatic event in which one’s life, physical integrity, or that of another, was severely threatened [13]. It is well recognised that police officers are exposed to such triggering incidents [14] at much higher rates than in many other careers and consequently are at increased risk for developing PTSD [15]. In support of this idea, previous studies have indeed quantified the degree to which typical police-related events, such as violence, death, or civil disorder, can be highly stressful [16,17]. Compared to the general population at an 8% rate of PTSD [18] and firefighters too [19], PTSD is higher in police officers at a rate of 9–15% [19,20]. This higher PTSD rate in police officers is coherent with the fact that their professional situations frequently entail risking physical and/or psychological harm, in cause promoting onset of the disorder.

BO is formally defined as a prolonged response to chronic stress at work [21], involving significant negative emotional and energy states that make it difficult to collaborate with colleagues and accomplish tasks at work [22]. Three principal dimensions are distinguished in the diagnosis of the syndrome: emotional exhaustion (i.e., frustration, lack of physical and emotional resources), depersonalization (i.e., cynicism, isolation, and loss of idealism) and thirdly, personal accomplishment (i.e., sense of achievement and value of work), which counterbalances the former two. Previous studies have found that as many as one-third of police officers display symptoms of BO [23,24] and nearly one-half display high depersonalization scores [24] and that these outcomes exceeded other emergency responders like firefighters or ambulance staff. This is in line with previous research that has shown PTSD, BO, and especially depersonalization and emotional exhaustion, to be strongly linked [25,26], suggesting that police officers may also have higher BO rates than in other careers. Moreover, within the framework of a multifactorial model, PTSD has been partly explained as the interaction between work-specific circumstances (e.g., confrontation to violence) and personality/psychological preparedness [19,27,28]. More broadly, across other careers, BO has also been partly explained via the interaction between professional situations, personality traits, and/or coping strategies [22,23].

A canonical personality trait model to which PTSD or BO has been linked to is the OCEAN personality model, developed by Costa and McCrae [29] and taken up again by John et al. [30]. OCEAN is comprised of five factors: O for openness, originality, and open-mindedness (i.e., curious personality); C for conscientiousness, control, and coercion (i.e., hardworking and persevering personality); E for extraversion, energy, and enthusiasm (i.e., sociable and dynamic personality); A for agreeableness, altruism, and affection (i.e., courteous, reliable and tolerant personality) and N for negative emotions, neuroticism, and nervousness (i.e., anxious, depressed, and emotionally unstable personality). Notably, previous research in police officers has found PTSD to be positively correlated with the neuroticism trait and negatively with the extraversion and conscientiousness traits [28,31]. Also, the emotional exhaustion and depersonalization dimensions of BO were found to be positively correlated with neuroticism while only depersonalization was negatively correlated with agreeableness, conscientiousness, and extraversion; finally personal fulfillment was negatively correlated with neuroticism and positively with the other four dimensions [23].

However, counting on personality traits alone, which are known to be essentially immutable, leaves little room for interventions and strategies in helping police officers manage serious mental disorders like PTSD and BO or avoid their acquisition. In this regard, encouraging the use of healthy and adaptive coping strategies may be a promising component of officer and career training programs. Coping involves an individual’s mechanisms for managing perceived internal and external stressors [32] and typically distinguishes emotion-focused coping, which allows one to face or reduce unpleasant emotions, from problem-focused coping, which allows one to change the origin of the stress or solve the problem [32,33]. Certain strategies may be characterized as more adaptive (e.g., active coping or positive reinterpretation) and others maladaptive (e.g., substance use or blame). In police officers, maladaptive coping strategies (avoidance, substance use) have been associated with higher levels of stress [27], PTSD symptoms [19], and BO [14,34], while adaptive strategies (problem-focused coping) correlate negatively with PTSD symptoms [15] and BO [14,34]. Although previous research suggests specific personality traits impacting the efficacy and choice of specific coping strategies [27,35,36] this has not been examined in police officers. Moreover, only one small study so far [37] has examined the mediation between PTSD and BO, specifically by mental rumination; but it did not take into account either coping strategies or personality. More broadly, several studies have examined the link between personality traits and the type of coping strategies utilized more versus less frequently as a function of them [27,35]. Notably, the extraversion trait was found positively correlated with positive distraction usage and negatively with denial and blame, while the latter two were positively correlated with the neuroticism trait [27]. However, it is important to note that research has also argued for the possibility that personality could have an interactional effect, that is, it could affect both the selection of the coping strategy and whether it is effective for the person in question [36].

To our knowledge, no study so far has examined altogether the principal stress dimensions (e.g., organizational stress, operational stress, PTSD and BO), in combination with personality traits and coping strategies, in police officers. We herein examine their relative importance via robust statistical and modelling analyses applied to a large sample (n > 1000) of police officers that were evaluated on these variables. The main objective of this study was to determine which stressors, personality traits, and coping strategies are relevant in the context of the development of PTSD and BO symptoms in police officers. Increasing our insight into such mechanisms may facilitate the development of prevention programs in police academies and forces. The second aim of this study was to determine if key officer profiles could be identified, based on observed outcomes tied to specific combinations of these variables. Such profiles could be used to screen and select recruits less prone to stress disorder in the occupation and to determine targeted mental health interventions.

## 2. Methods

### 2.1. Participants

All participants were police officers who, at the time of data collection, possessed a Swiss Federal Patent (or equivalent, for the older participants) and were working in the police forces of the French-speaking regions within Switzerland. The officers worked in a variety of suburban or municipal police forces and services.

### 2.2. Materials and Design

The data of this cross-sectional study were collected digitally by means of a fully anonymised online questionnaire sent by email to approximately 4250 eligible officers originating from 15 different police forces. For each respective police force, the email calling for participation in the study was relayed by the command post or the psychological services unit of the force. Prior to beginning the questionnaire, each participant first needed to sign an electronic consent form. The study received ethical authorization from the Ethics Committee of UniDistance Suisse on 17 April 2019.

The first part of the questionnaire consisted of questions on socio-demographics and occupational stress (see Supplementary data) and the second part the following standardized questionnaires: the Impact of Event Scale—Revised [38,39], the Maslach Burnout Inventory [22,40], the Big Five Inventory [30,41], the Brief COPE [42,43], the Hospital Anxiety and Depression Scale [44,45] and suicide ideation from the Beck Depression Inventory—II (BDI-II, [46]).

#### 2.2.1. Primary Outcomes

The Impact of Event Scale—Revised (IES-R) [38,39] is a 22-item self-report scale measuring symptoms of PTSD, in which herein the French adaptation was used [38,39]. The scale is divided into 3 subscales: intrusion, avoidance and hypervigilance. The participant is asked to identify a specific stressful event by indicating how much difficulty the event has caused in the past 7 days. Responses are given on a 5-point Likert scale ranging from 0 (“not at all”) to 4 (“extremely”). The sum of the three subscale scores determines a composite PTSD score for which the typical clinical threshold for PTSD presence is a score ≥ 33 [47]. Note that scores ≥ 24 indicate a substantial clinical concern [48] and scores ≥ 37 have been associated with immune system suppression, even 10 years after the traumatic event [49]. Herein, the Cronbach’s α values obtained were respectively 0.92, 0.83 and 0.85 for the intrusion, avoidance, and hypervigilance subscales.

The Maslach Burnout Inventory (MBI) [22] is a 22-item self-report scale measuring BO, in which herein the French adaptation was used [40]. The scale is divided into 3 subscales: emotional exhaustion, depersonalisation and personal accomplishment. The participant is asked to determine how often he or she experiences certain situations. Responses are given on a 7-point Likert scale ranging from 0 (“never”) to 6 (“every day”) [22,40,50]. BO positivity is determined by a nonlinear combination of the scores, specifically if either of the following conditions are satisfied: (i) exhaustion and depersonalisation are both ≥ 18 or (ii) exhaustion is ≥ 18 and accomplishment is ≤ 34. Herein, the Cronbach’s α values obtained were respectively 0.87, 0.73, and 0.80 for the exhaustion, depersonalisation and accomplishment subscales.

The Big Five Inventory in French (BFI-Fr) [30,41], herein referred to as B5, is a French adaptation of the classic, 45-item self-report scale assessing each of the Big Five personality dimensions of the OCEAN framework, previously discussed: Openness, Conscientiousness, Extraversion, Agreeableness and Neuroticism. The participant is asked to determine the extent to which he or she agrees with statements. Responses are given on a 5-point Likert scale ranging from 1 (“strongly disagree”) to 5 (“strongly agree”). Higher resultant scores in each subscale reflect stronger presence of the personality trait. Herein, the Cronbach’s α values obtained were respectively 0.78, 0.78, 0.85, 0.75 and 0.84 for the openness, conscientiousness, extraversion, agreeableness, and neuroticism subscales.

The Coping Orientation to Problems Experienced, abbreviated version (Brief-COPE) [42], herein referred to as BCope, is a 28-item self-report scale evaluating the extent to which 14 different coping strategies are utilised: Acceptance, Active Coping, Blame, Behavioral Withdrawal, Denial, Distraction, Emotional Expression, Emotional Support, Humour, Instrumental Support, Planning, Reinterpretation Positive, Religion, Substance Use. Herein the French adaptation was used [43]. Responses are given on a 4-point Likert scale ranging from 0 (“not at all”) to 3 (“always”) and higher scores reflect a higher tendency to implement the corresponding coping strategies. A Cronbach’s α of 0.85 was herein obtained for this scale.

The Police Stress Questionnaire (PSQ) is a short version, 10-item self-report scale, consisting of two subscales that separately measure operational and organizational stress, specifically with regard to the police work environment. The PSQ was developed herein in the scope of addressing the lack of formal questionnaires currently available that measure police operational and organisational stress, as compared to more typical careers. The PSQ herein is not aimed to replace or be preferred over longer or formal, fully-validated alternatives of police job stress that may come to term. Here the PSQ was conceived as a short or abridged, scale in order to encourage overall officer response credibility in an already long questionnaire, due to the high multivariate scope of the study. The PSQ was comprised of items based on a review of directly related published studies and questionnaires that have considered police operational organizational and personal stressors [6,9,51]. Specifically, the principal stressors described in these studies were translated to questions in French. Then a subsample of francophone police officers was surveyed to rate the top five most pertinent stressors operationally and organisationally. After compilation of the results, for each subscale, the top-five stressors were retained in order to compose the final questionnaire, resulting in 10 items total. For each subscale, the resultant items were nonredundant in measurement or orthogonal respectively. Lastly, one additional item that directly asked about their degree of work-life balance was also included. The list of items are provided in the Supplementary Materials. For all items, responses were given on a 5-point Likert scale ranging from 1 (“strongly disagree”) to 5 (“strongly agree”) with the statement provided. The Cronbach’s α values obtained were respectively 0.62 and 0.63 for the operational and organizational stress subscales. Note that these Cronbach’s α values for the PSQ are consistent with the lower scores that are typically found in the literature for such short scales with non-redundant items [52]. For example, in the popularly-used Ten Item Personality Inventory (TIPI) scale, containing only 2 items for each of five personality traits, low-to-moderate Cronbach’s alphas (α = 0.40–0.68) have been established and have been frequently replicated in subsequent studies [53,54,55]. Herein, in a confirmatory factor analysis of the short PSQ questionnaire, the typical indices for goodness-of-fit were satisfied (CFI, IFI GFI and AGFI > 0.90, TLI = 0.89).

#### 2.2.2. Secondary Outcomes

The Hospital Anxiety and Depression Scale (HADS) [44] is a 14-item self-report scale, consisting of two subscales that separately measure anxiety and depression. Herein the French adaptation was used [45]. The participant rates how often he or she experiences different feelings. Responses are given on a 4-point Likert scale ranging from 0 to 3, where the scale description may vary according to the question. The typical clinical threshold for anxiety presence is a score ≥ 8 and likewise for depression and a total score ≥ 11 may reflect an adjustment disorder in general albeit due to anxiety or depression [56]. Herein, the Cronbach’s α values obtained were respectively 0.78 and 0.73 for the anxiety and depression subscales.

The item on Suicidal Ideation (BDI-II) from the Beck Depression Inventory [BDI-II; 46], which has shown to have good predictive validity in previous studies [57], was incorporated into the questionnaire and translated into French. The response to this item is coded on a 4-point Likert scale as follows: 0 (“I don’t have any thoughts of killing myself”), 1 (“I have thoughts of killing myself, but I would not carry this out”), 2 (“I would like to kill myself”) and 3 (“I would kill myself if I had the chance”).

The following demographic variables were collected and included in the analyses: age, gender, relationship status, number of children under 18 in the household, pre-police academy education, number of years of service, current work position and police force.

### 2.3. Data Pre-Processing and Statistical Analysis Approach

The analytical approach here utilized a similar methodology as used in [58]. First, descriptive statistics and frequency counts (e.g., positivity) were calculated along the relevant variables and questionnaires (see Supplementary Table S1). Then, in preparation to satisfy modelling and statistical criteria (e.g., normality, homoskedasticity, linearity for regression), noncategorical variables were normalized via the Yeo-Johnson transformation [59] and scaled. Two-sample *t*-tests were then calculated based on pathology presence, for all continuous variables and *p*-values were Holm-Bonferroni corrected [60] for multiple comparisons.

A backward stepwise linear multiple regression model (*statsmodels* package in Python, version 0.14) was realized to identify the most pertinent variables that predict the IES-R total score and similarly with a logistic regression approach for predicting BO positivity (MBI scale). For each application, first in the full model with all variables, approximately 5–15% of the data were filtered as outliers based on Cook’s Distance [61] values that exceeded two times the mean value. Note that this percentage is coherent with the regression modelling literature where up to 10–20% of a sample may be acceptably identified, depending also on the characteristics of the data set and the outlier detection statistic used (for exemple, see [62,63,64,65]). Then, predictor variables in the model were eliminated recursively by which removal would most improve the Akaike Information Criterion, AIC [66]. For both resultant models, the necessary assumptions and diagnostics were rigorously evaluated and these are provided in Section 3.

Lastly, a principal components analysis and clustering of the participants was performed (*scikit-learn* library in Python) to obtain additional insights into the police officer sample and identify potentially insightful officer profiles. The observed Hopkins *H* = 0.71 for the data, which supports a strong tendency to cluster. Specifically, the principal component decomposition of the data (N = 5 dimensions) was submitted (see [67]) to a spectral clustering algorithm [68]. In contrast to the canonical clustering approaches, such as *k*-Means, spectral clustering is rooted in graph theory; it makes limited assumptions about the shape/form of the clusters and rather treats cluster identification as a graph neighborhood optimization problem. Using this approach, an optimal clustering result that balanced interpretable parsimony and performance (e.g., cluster separation and compactness) was obtained at *K* = 3 clusters. The performance diagnostics for this final model are provided in Section 3.

## 3. Results

### 3.1. Participants

A total of 1073 police officers fully completed the questionnaire (25% response rate). One participant was removed based on providing an age of 16 years old; otherwise, the youngest officer included in the study was 22 years old. As for age, 12% of officers were between 22 to 29 years old, 37% were 30 to 39 years old, 30% were 40 to 49 years old and 21% were 50 to 65 years old. Most officers completed up to vocational school level 1 (62%), others up to high school (18%) and the remainder either vocational school level 2 (8%), university (9%) or other schooling (3%). Twenty-two percent of officers were female. Professionally, 36% of officers were emergency police, 31% judicial police, 13% community police, 6% administrative, 5% traffic, 5% special forces, and 3% dispatch center. Nearly one half of officers, 49%, had at least one child. The interquartile range for years of service was 7 to 23, with a median of 15 and mean (SD) of 15.6 (10.1).

### 3.2. Clinical Symptoms

**Figure 1:**
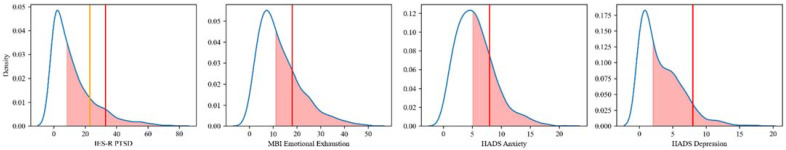
Distributions of pathological scores and cutoffs (vertical lines) for each of the scales (left to right) IES-R PTSD (clinical concern ≥ 24 in orange; 16% and positivity ≥ 33 in red; 9%), MBI Emotional Exhaustion (≥18), HADS Anxiety (≥8), HADS Depression (≥8) Scales. The red shaded area denotes where the upper 50% of police officers are situated (the median and above). The area to the right of the vertical lines designates pathology presence (e.g., ≥threshold).

The profile of clinical symptoms of the police officer sample is summarized in Table 1, in which for each scale, the mean (SD), interquartile range (IQR), max value, score ≥ clinical threshold to determine pathology presence and the % of individuals satisfying this threshold is provided. Figure 1 then provides a visualization of this information, showing the distribution for each of the scales and their respective, recommended clinical thresholds (red vertical lines). This visualization helps to demonstrate the degree of continuity in the presence of each of the different pathologies (pre-, partial, full) and that this population presents a consequential profile of pathological symptoms, with a likely risk for exacerbation on the individual level, in light their continued occupational demands. The red shaded area denotes where the upper 50% of police officers are situated (the median of the sample and above).

Starting with PTSD, 16% of officers satisfied the criteria for substantial clinical concern, also known as a state of pre-diagnosis or partial PTSD symptoms (score ≥ 24), 9% for the disorder (score ≥ 33) and 6% for PTSD-induced immune system suppression (score ≥ 37). Proportional to numbers of officers by position occupied, traffic police, dispatch center and community police were highest in PTSD positivity at 17%, 14% and 12% respectively; the lowest were judicial police at 7%.

Next regarding BO, approximately 16% satisfied the criteria for the disorder, which involves several possible combinations of the three MBI subscales. The Emotional Exhaustion dimension is the principal negative dimension used in these combinations. In this respect, 25% of police officers satisfy the criteria (score ≥ 18); in which 23% of these officers may be considered severely exhausted (score ≥ 30). Proportional to numbers of officers by position occupied, dispatch center, special forces, emergency and judicial police were highest in BO positivity at 29%, 19%, 17% and 17% respectively; the lowest were administrative police at 10%.

In respect to the HADS Anxiety and Depression scales, respectively 25% of officers satisfied the criteria for a clinical anxiety disorder (score ≥ 8) and 9% for depression (score ≥ 8). In respect to the total HADS score (Anxiety + Depression), impressively 28% of officers have a score ≥ 11, signaling a major prevalence of strong emotional disturbance.

Finally, as many as 14% of police officers expressed having at least thought of committing suicide (score ≥ 1). However, only 0.3% of police officers admitted a desire to commit suicide (score ≥ 2).

### 3.3. Variables Associated with a Lower Clinical Symptoms

The previous section demonstrated a real issue of different mental or emotional disturbances being present in the police officer sample. In order to get an idea of the variables linked to pathology presence respective to each of the measures and thresholds in Table 1, two-sample *t*-tests were performed between the pathological group and non-pathological group for every continuous variable. The *t*-values are provided in Table 2, for which the *p*-values (Holm-Bonferroni corrected for multiple comparisons) are reflected by asterisks. The variables in the table are organized based on most significantly negative (less present in the pathological group) in descending magnitude; these are above the first horizontal line and below: most significantly positive (more present in the pathological group) likewise in descending magnitude. For brevity, variables that did not exhibit a significant difference for any pathology are not included in the table.

Based on these results, one can observe that positive reinterpretation and humour are among the strongest coping strategies linked to absence of each pathology; then acceptance and active coping were linked to BO absence but not for most of the other pathologies. Emotional expression was solely linked to depression absence.

In respect to personality traits, agreeableness was linked to absence for all pathologies except for PTSD (but not far from significance, as *t* = −2.57, did not survive the Holm-Bonferroni correction). Next, conscientiousness and extraversion were linked to the absence of BO and mildly to the absence of anxiety and depression.

Linked to pathology presence, the neuroticism trait was the strongest out of all types of variables and for all pathologies; most markedly, neuroticism was linked to the presence of anxiety disorder (*t* = 19.66) and then BO (*t* = 11.11). The next strongest variables were organizational stress and life imbalance, associated with all pathologies. Organizational stress was most strongly linked with the pathological presence of BO and anxiety and most weakly linked with the presence of PTSD. Life imbalance was also most strongly linked with the presence of BO and depression in second. Organisational stress was significant for all pathologies as well, but was much less strong in average magnitude as compared to the former three variables mentioned.

In respect to maladaptive coping strategies, behavioural withdrawal, blame, substance abuse and denial were the most strongly associated with pathology presence for all pathologies and were fairly balanced in magnitude across them. In contrast, the distraction strategy was only significant for the presence of PTSD and anxiety disorder, which was also the case for the religion and emotional support strategies.

In respect to demographic variables, increasing age, more years of service and male police officers were all associated significantly with the presence of suicide ideation, but not any other pathology.

### 3.4. Predicting Post-Traumatic Stress Disorder (IES-R)

The previous two-sample *t*-test analyses provided a useful identification of the major variables linked to the presence of each pathology. However, they do not take into account the continuity of the information, that is, how variables may be linked to increases or decreases in the degree of pathology, even if an officer has not necessarily reached clinical levels for example. They also involved independent comparisons and do not take into account how variables may combine or be redundant in predicting a pathology.

This more detailed level of description is possible with a multiple regression analysis, which the results for predicting total PTSD scores (IES-R scale) are provided in Table 3. These results are based on a significant predictive equation *F*(24,875) = 29.88, *p* < 0.001 that provided an *R*^2^ and adjusted *R*^2^ of 0.45 and 0.44 respectively. Also, the diagnostics for appropriate model fit were assessed and satisfied (e.g., see [69,70]). Specifically, residual normality was assessed and satisfied by the Komolgorov-Smirnov Jarque-Bera, Anderson-Darling and Omnibus tests (all *p* > 0.05); homoskedasticity by the Breusch-Pagan, White and Goldfeld-Quandt tests (all *p* > 0.05); absence of multicollinearity was verified and satisfied by small observed variance inflation factors for each predictor (maximum = 3.2); multivariate normality was satisfied by the Yeo-Johnson transformation; absence of autocorrelation was verified and satisfied by the Ljung-Box and Lagrange Multiplier tests (both *p* > 0.05); and linearity verified and satisfied in the Rainbow and Ramsey tests (both *p* > 0.05).

The results in Table 3 demonstrate that in the context of an integrative model that may eliminate redundancies (recursive elimination), only three main variables predict lower PTSD scores: the openness personality trait, and humour and active coping strategies. In contrast, a number of variables are significantly associated with successfully predicting higher PTSD scores, the most mentionable in ranked (*t*-value) order are operational stress, substance abuse, behavioral withdrawal, distraction, religion, emotional support, the neuroticism personality trait, and denial.

### 3.5. Predicting Burnout (MBI)

As clinical BO pathology is determined by several logical comparisons between the MBI subscales and not a threshold of the total sum score, multiple linear regression is not pertinent for identifying the most relevant predictors, rather, a statistical implementation of multiple logistic regression is, for which we provide the results in Table 4.

A significant equation was found *F*(23,993) = 40.07, *p* < 0.001 (model deviance compared to intercept-only model) that appropriately satisfied the standard diagnostics (e.g., see [71]), such as the Likelihood Ratio Test and the Lagrange Multiplier Score Tests for overall model fit (*χ*^2^(2) = 921.6, *p* < 0.001 and *χ*^2^(23) = 602.5, *p* < 0.001 respectively); and the Wald Test (*χ*^2^(4) = 148.2, *p* < 0.001), satisfied based on the joint hypothesis of the inclusion of the top-four most significant predictor variables (PSQ Stress Organisational, B5 Neuroticism, PSQ LifeImbalance, BCope Active). In agreement, the AIC (537.7 vs. 1413.3) and BIC (−6394.4 vs. 1418.2) values indicated clear preference over a null model that is intercept-only (see [72]). Pseudo-*R*^2^ values were obtained for the model that are considered strong (i.e., values between 0.6 to 1.0): McFadden *R*^2^ = 0.65, Nagelkerke *R*^2^ = 0.79 (see [73]) and an appropriate AUC score of 0.86 was obtained (see [74]); the true negative detection rate (BO absence) was 87% and for true positives, 74%.

The results in Table 4 demonstrate that the strongest predictors of the absence of BO were the active and positive reinterpretation coping strategies. Following them were the agreeableness and conscientiousness personality traits. Finally, the acceptance coping strategy was the last significant absence predictor. In contrast, the strongest predictors of BO presence were organizational stress, the neuroticism personality trait and life imbalance. Following them were three seemingly dysfunctional coping strategies: behavioral withdrawal, distraction, and substance abuse.

### 3.6. Psychological Profiles of Police Officers

**Figure 2:**
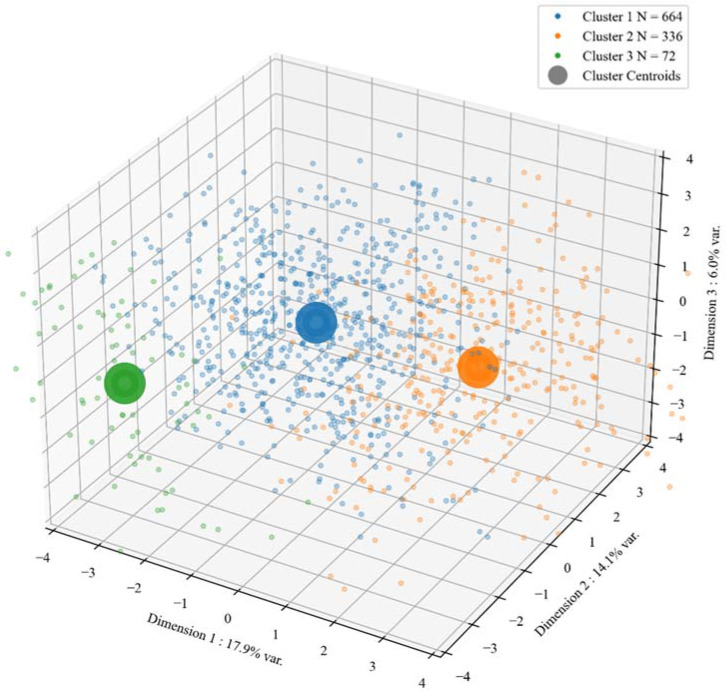
Clusters of the police officers obtained from an optimized spectral clustering algorithm. Each point represents an individual officer’s values on the first (*x*-axis), second (*y*-axis) and third (*z*-axis) principal components of the data (Dim 1, Dim 2, Dim 3). The cluster centroids are calculated as the mean value of the cluster for each of the three dimensions. This diagnostic demonstrates the satisfactory result of within-cluster cohesion and between-cluster separation with respect to the three strongest principal components, as per the total variance they explained (17.9%, 14.1% and 6.0%, respectively).

The clustering analysis revealed three well-defined clusters that balance interpretative parsimony and performance (separation and compactness). The resultant diagnostics were as follows: Davies-Bouldin score = 1.47 ([0, ∞+], lower values preferred), Calinski-Harabasz score = 241.11 ([0, ∞+], larger values preferred), Dunn Index = 1.49 ([0, ∞+], larger values preferred and average Silhouette score = 0.19 ([−1.0, 1.0], larger values preferred). The satisfactory result of within-cluster cohesion and between-cluster separation with respect to the three strongest principal components, as per the total variance they explained (17.9%, 14.1% and 6.0%, respectively) is visualised in Figure 2.

**Figure 3:**
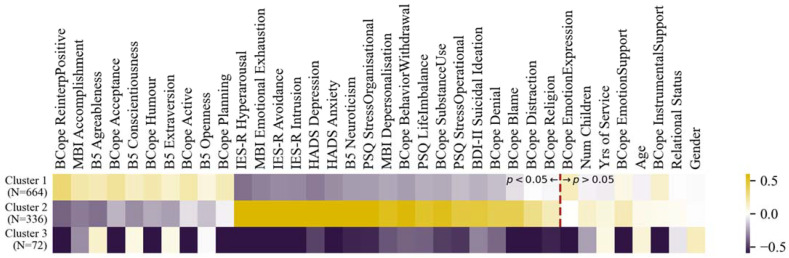
Hierarchical clustering analysis: Mean values of each variable (after Yeo-Johnson transformation and standardization) for each cluster. Variables are sorted based on the most significant positive differences between cluster 1 and 2, most significant negative differences, then non-significant differences. All pairwise significance tests between the clusters are provided in Table S2 in the Supplementary Materials. Note: Bcope = The Coping Orientation to Problems Experienced Inventory, B5 = Big Five Inventory in French, IES-R = Impact of Event Scale–Revised, MBI = Maslach Burnout Inventory, HADS = Hospital Anxiety and Depression Scale, BDI-II = Beck Depression Inventory, PSQ = Police Stress Questionnaire; see Section 2 for a detailed explanation.

Based on the cluster memberships derived from the algorithm, the cluster means for each of the variables collected (calculated after after normalisation and standardisation) are provided in Figure 3 and they are sorted based on the strongest significant positive differences, then negative significant differences, between the first two clusters (*p*-values Holm-Bonferroni corrected). For a full table of pairwise significance tests between the clusters, see Table S2 in the Supplementary Materials.

The clustering analysis may also permit one to take a look at the traits and strategies associated with reduced pathologies together, instead of separate analyses of each pathology. For example, based on the clustering analysis in Figure 3, for the majority of police officers who may be faced with stressful situations, the difference between Clusters 1 and 2 show that positive reinterpretation, sense of accomplishment, acceptance, sense of humour, active coping and planning were associated with the absence of clinical levels of the different pathologies and their subscales, such as for PTSD (IES-R), BO (MBI), anxiety (HADS), depression (HADS) and suicide ideation (BDI-2). The personality traits most associated with low levels of these were agreeableness, conscientiousness, and extraversion (B5). These results corroborate or summarise well those found in the previous separate analyses such as in the two-sample positivity *t*-tests and the separate linear and logistic regressions on PTSD and BO. Cluster 3 concerns a small sample who are insensitive to any variable except that they have a positive personality profile.

## 4. Discussion

The results of the present study that are based on a robustly large sample of police officers (N > 1000), confirm that police personnel present a clinical profile that is of high risk for various mental disorders, including notably PTSD and BO (see Table 1 and Figure 1). These disorders, PTSD and BO, were the primary focus of this study. The rates of partial (16%) and full PTSD (9%), based on the IES-R, found in the sample of the present study is comparable to that of a systematic review that found a median and mean rate of 9.2% and 14.9% respectively [75], which are each higher than the prevalence in the general population [18]. Regarding the percentage of police officers with BO, our finding is more than half as high as in another large study by De la Fuente Solana et al. [23]. This difference could be explained by the fact that Swiss and Spanish police forces have different scopes of operating (e.g., national vs. county police levels) and organisational means (e.g., number of staff or materials). As for anxiety and depression, the prevalence rates that were herein found were within the range of those previously found within a systematic review including 15 studies on depression and 4 on anxiety [76], with the departure from some studies that here the anxiety rates (25%) were over twice those as depression rates (9%). The prevalence of suicidal ideation in our sample (14%) is half that of Guerrero et al. [77] but well above those (4.6%) in the Soravia et al. [19] study as well as the general population (9.2%) [78]. These differences could be explained by the questionnaires used in these studies as having different constructs.

### 4.1. Identification of Risk and Protective Factors for PTSD and BO through Integrative Analyses

In the context of an integrative model predicting current PTSD levels, the results marked the importance of neuroticism as a major risk factor and openness as a protective factor. These findings are supported by other correlational studies [28,31]. Our results also demonstrated, as in other studies [15,19], that adaptive strategies (e.g., humor or active coping) tend to predict lower PTSD symptoms and can therefore be seen as protective factors for PTSD, other so-called maladaptive coping strategies (e.g., distraction, denial, or substance abuse) emerge as risk factors for PTSD. The link between PTSD and operational stress is theoretically sound in this population, given that most items in the operational stress scale can be directly linked to stressful event exposure. For example, “I regularly respond to urgent interventions”, “I am regularly faced with physical threats”, etc., many of which said events may involve violent situations (e.g., aggressive behavior, domestic disputes, motor accidents, death), which may be frequently appraised by the officer as an imminent threat to their personal survival/well-being or that of others.

In respect to BO, our research also highlights the importance of the interplay of certain personality traits as a risk or protective factors. Indeed, compatible with the findings of De La Fuente Solana [23], agreeableness and conscientiousness seemed to emerge as protective factors when neuroticism appears as a risk factor for BO. One explanation would be that individuals with salient neuroticism traits would be more prone to negative emotions, preventing them from finding resources to cope with BO, while people showing agreeableness traits would have better social support from their surroundings, allowing them to reduce occupational stress [79]. The results show, in a manner similar to other studies [14,34,80], that coping strategies can be protective or risk factors for BO depending on whether they are adapted or maladaptive. In addition, stress: whether operational, organisational, or related to family/work life balance, also appears as a risk factor, corroborated by other studies [34,81]. In respect to occupational stress, our modelling results revealed that BO is primarily influenced by organisational stress rather than operational stress, conversely to PTSD. Indeed organisational factors, including work recognition and the quality of one’s relationship with supervisors, have been previously identified as significantly influential factors of BO [82]. Also consistent with our finding, is a previous study that showed high operational stress exposure was not significantly linked to increased burnout severity [83].

These results can also be viewed as congruent with Appraisal Theory [84,85], which argues that over the frequency of stress exposure, what is more determinant in pathology development is rather the individual’s interpretation of the stressors and their personal relevance (known as primary appraisal) and in second place, the individual’s evaluation of their available resources and options for coping with the situation (known as secondary appraisal). In this light, coping strategies and organizational factors may be interpreted as part of an individual’s secondary appraisal functioning.

These modelling results were corroborated by the two-sample *t*-tests herein on the different pathological variables. Rather than taking into account the continuous value of the pathological variable (e.g., IES-R PTSD score) and many predictor variables simultaneously, as in a linear regression model, the two-sample *t*-tests compared, for each variable separately, the values of the pathological versus non-pathological group. These groups were determined by whether the pathological variable exceeded the recommended clinical threshold (see Table 1 and Table 2). For example, in terms of corroborating results in these tests: positive reinterpretation, which is positively correlated with humour (Pearson *r* = 0.39, *p* < 0.001), was found adaptive in respect to the presence/absence for PTSD and BO, as well as for each of the other pathologies. In respect to Anxiety and Depression and in line with other studies, the personality traits of agreeableness and conscientiousness were here identified as protective factors for both [86], while extraversion was specifically protective for Depression. In terms of coping strategies, acceptance was specifically adaptive for Anxiety and for Depression, active coping [87] and emotional expression [88,89]. Similar to other studies [90,91,92], the trait of neuroticism is a major risk factor for both affective pathologies, though more strongly for Anxiety. In respect to suicidal ideation (SI), neuroticism was again identified as a significant risk factor, which is likewise line with other studies [93,94] and agreeableness seemed to act as a protective factor [93]. Furthermore, several maladaptive strategies were identified (i.e., religion or avoidance coping, substance abuse) [94,95], while positive reinterpretation was found as an adaptive strategy. Similar to the other mental disorders previously discussed organizational stress [5,12] and life imbalance [96] were tied to SI. However, unlike PTSD and BO, age (i.e., older) [93,96] and gender (i.e., male) [96] were identified as risks factors for SI.

### 4.2. Identification of Police Profiles at Risk Versus Resilient to PTSD and Burnout

Similar to the profiling of people at risk of committing murder (e.g., [97]), our study sought to also identify profiles, yet through a positive light and in the service of police officers: specifically, which police officers tend to manage best the notably difficult and stressful conditions of their job? The strength of the present study involved the identification of psychological and coping tendency profiles of officers, which could be used instrumentally to offer innovative mental health disorder prevention and mitigation programs. It is evident that police officers are exposed to significantly higher levels of stress. Fortunately, a large proportion of the force may be identified as psychologically resistant; while select others may be spared by only a weak regular exposure to stressors. In contrast, it is crucial that the remainder of the police force who are at high risk of mental disorders (in our large sample, approximately one-third), benefit from interventions in the interest of career longevity and personal health.

Indeed, our clustering analyses highlighted two clearly distinct psychological profiles in this respect, in which approximately two-thirds of police officers possessed a psychological profile resilient to stress, with low risk of PTSD and BO and one-third vulnerable to stress with high symptoms of PTSD and BO. The resilient officers were more agreeable, conscientious, extroverted, and open to experience on average. They tend to use positive reinterpretation, acceptance and planning as coping strategies. Conversely, the police officers at high risk for stress-related disorders such as PSTD, BO, suicide ideation, anxiety and depression displayed strong traits of neuroticism and used more frequently substance abuse, behavioral withdrawal, blame and denial as coping strategies.

A unique contribution of the clustering approach is its capacity to explain how multiple pathology measures can be associated with different variables, rather than one pathology independently at a time, as in the previous regression or *t*-test analyses. In consequence, variables that may relate to multiple pathologies may be more strongly identified as significantly different between the clusters. Interestingly, the variables that are decisively different between the stress resilient (Cluster 1) and stress vulnerable (Cluster 2) were strongly corroborated by the previous regression and *t*-test results. For example in the previous analyses, the openness personality trait was linked with lower PTSD and agreeableness and conscientiousness traits with lower BO, which were also found in the stress resilient Cluster 1. Neuroticism was likewise found in all the previous analyses as strong risk factor for the pathologies and was as the strongest significantly different risk factor between Clusters 1 and 2. Not found in the regression, high extraversion was found in Cluster 1 though coherently in the *t*-tests it was most strongly linked to lower rates of depression and burn out, which is consistent with the pathological profile of Cluster 2. Similar consistencies between the analyses can also be found with regard to both the adaptive (e.g., positive reinterpretation, humor, active coping) and maladaptive coping strategies (e.g., substance use, blame, denial). The clustering analyses newly highlighted the planning strategy to differentiate Cluster 1 and 2, which may link to multiple pathologies but not strongly enough to one specific pathology to be significant in the previous analyses. Furthermore, the emotional expression strategy was not significant between Clusters 1 and 2, which can be explained by its negative association with lower depression rates (*t*-tests), while having a positive association with PTSD (regression, also for emotional support). To better disentangle the long-term efficacy of these emotion-centered strategies for PTSD and BO, further data is needed such as from a longitudinal study design.

Finally, the presence of Cluster 3 suggests that subsequent investigation may also be needed to fully understand why some police officers are not touched by such pathologies, despite their lack of implementing different positive coping strategies. The results show that their PSQ occupational stress levels are lower than the other clusters, but these differences are not significant if specifically compared to Cluster 1. The simplest explanation may be that these officers are less-frequently exposed to traumatic or repeated anxiety-producing events and hence are generally not stimulated to call upon them. Including an additional questionnaire with more specific questions regarding regular anxiety-provoking/disturbing events may provide data allowing for further differentiation of these clusters to more refined levels or further improve pathology prediction in modelling analyses in future studies. Nonetheless, importantly Cluster 3 has absent the maladaptive coping strategy of blame, which is potentially a key element that could be used to explain the cluster, as it could be linked to their model of personal responsibility for job outcomes (e.g., failure, civilian or partner death). Alternatively, the cluster could also be explained by a trait of much lower susceptibility to arousal or emotional impact, for which an additional measurement scale may be necessary.

### 4.3. Relationship of Findings to Non-Police Populations

Our analyses that confirmed the relationship of coping strategy influence on burn-out in police officers is consistent with previous burnout findings in other populations. For example, a systematic review of 85 studies and 261 predictors [98] demonstrated a positive impact of adaptive coping strategies and leisure activity (here PSQ Life Imbalance) and conversely, a negative impact of job demands (here PSQ Stress Organisational). In particular, active coping and acceptance have been highlighted in many studies as adaptive strategies and the use of psychoactive substances as maladaptive [99,100]. Most of the maladaptive coping strategies of PTSD that we have highlighted (behavior withdrawal, distraction, denial) corroborate the cognitive model of posttraumatic stress disorder [101] which identifies such avoidance tendencies as a substantial factor in maintaining traumatic memories [102].

However, other coping strategies, such as religion, emotional support and emotional expression have been described in previous studies as adaptive [103] whereas in our study they are correlated with PTSD presence. Several explanations can be offered to explain this distinction: such as whether specifically the police officer population interacts differently with strategy choices in these domains; whether the questionnaire used to measure these coping strategies (BCope) was not the optimal choice and should be reexamined; and/or whether these three coping strategies should be interpreted rather as a renunciation of an internal locus of control. For example, a said renunciation would correspond to a lack of active coping, which we found as protective for PTSD and BO and can be linked towards an individual’s adoption of a model of external locus of control, which has been identified as a risk factor for PTSD [104,105].

Altogether, as one of the only studies to date to have jointly assessed a vast number of dimensions known as highly-relevant to PTSD and BO (personality, coping strategies organisational and operational stress, demographics) within an integrative model using data-driven approaches and a robustly large sample, our study makes a theoretical contribution towards understanding PTSD and BO through a triangular model comprised of the pathologies’ contextual (Occupational Stress), reactional (Coping Strategies) and psychological (Personality Structure, Affect, Comorbidity, Demography) dimensions.

### 4.4. Meaning of the Study and Possible Implications

The results of the present study suggest that the screening of personality profiles and coping tendencies could be instrumental in the improved selection of recruitment candidates for the police academy, in order to identify individuals with a lower risk of developing PTSD or BO on the job. Also, targeted interventions for current employees that need them most and would respond positively, could be conceived, based on their personality and coping strategy profiles. Not only the coping literature, but also many publications on emotion regulation suggest that certain strategies may have more positive long-term effects compared to others [106]. Respective to their regular use in a long-term perspective, certain strategies can be considered as adaptive (cognitive reappraisal, acceptance), while others can be seen as maladaptive (avoidance, rumination, expressive suppression). Moreover, coping strategies focused on positive emotions, such as positive reinterpretation and humor, can be used to limit negative emotions by increasing positive emotions [107] and have been associated with better recovery from trauma [108]. The results from the current study shed light on potential key adaptive strategies that could be emphasized during academy training [109,110] or mid-career [111].

*Humor*: This coping strategy could offer an important medium to reinforce team cohesion and express support to team members [112,113,114] and crucially, mitigate the perception of difficult experienced events [113]. The beneficial effects of humor as a coping strategy have also been confirmed by other different studies. Indeed, women affected by armed conflict in Georgia who used humor as a coping strategy had fewer symptoms of post-traumatic stress [115]. Furthermore, several studies have found that exchanging jokes can create a positive work atmosphere and therefore protect against BO [116,117,118].

*Positive reinterpretation*: It is well-established that emotional and behavioral responses to an event are based on the individual’s appraisal of that event [84] or alternatively, the level of stress to an event depends on the cognitive evaluation of that stressor [85]. Modifying the cognitive assessment of a stressor (for example, reinterpreting civilian aggression as a product of an unjust society rather than as personally targeting the specific police officer) is the basis of most stress and anxiety management interventions today and is hence closely tied to positive reinterpretation. Cognitive-Behavioral Therapy (CBT) is currently the predominant effective therapy used by clinical psychologists for the treatment of anxiety disorders and stress [119]. These psychotherapies are essentially based on cognitive reappraisal and behavioral activation and for example, have been found effective in the treatment of depression [120]. Cognitive reappraisal has been associated with less post-traumatic stress symptoms [121] and enhanced exposure efficacity in PTSD treatment [122].

*Acceptance and Active coping*: Both beneficial coping strategies found in our analyses, acceptance and active coping, are directly relevant to one of the latest branches of CBT, known as Acceptance and Commitment Therapy (ACT). For example, ACT has been found effective for treating issues of BO [123], PSTD [124], alcohol use disorders [125] and suicidal ideation [126] in veterans. ACT may be also highly relevant in flagging the dissonance that could arise between values and commitment behaviors established with the officer and cases in which their organization may force them to behave oppositely and hence engendering stress [127,128]. Finally, as ACT is recognized as a transdiagnostic approach, training police officers with ACT may be pertinent both for the prevention and treatment of mental disorders.

### 4.5. Strengths, Limitations and Future Work

A notable strength of our study concerns its sample size. Indeed, the majority of current studies available on police officers involve only a few hundred participants (except [34,129]). This large sample can allow for a notable level of robustness and reliability. Although the participation rate, which was on a voluntary basis, may seem low, rather the opposite could be argued: collecting so many responses to such a long series of questionnaires constitutes a consequent return. This detailed amount of data collection favorably positioned us to be able to take into account a very large number of relevant factors simultaneously, compared to other studies which may have more blind spots. Nonetheless, it would have been worthwhile to also assess other variables such as traumatic anamnesis with the Trauma History Questionnaire [130] or stressful life events with the Life Event Stress Scale [131]. In the context of increasingly highly multidimensional (multivariate) data, path analyses and more advanced modelling approaches may importantly serve in a future study on this data to better understand the nuances and intermediary relationships between the large number of variables involved.

We utilized validated scales which are the most commonly used in the scientific literature of this domain. For some cases however, it would have been relevant to seek more modern questionnaires. For example, Maslach’s model of BO and MBI scale have been heavily criticized, particularly on its dimension of depersonalisation [132]. Today, the Job Demands-Resources model (JD-R; [133]) is increasingly recognized. Therefore, either the Burnout Assessment Tool (BAT; [134]) or the Oldenburg Burnout Inventory (OLBI; [135]) could replace the MBI in a future study.

While our findings regarding humor are interesting, the choice of the scale assessing humor might also be considered a limitation. For instance, the current scale does not permit any conclusions to be drawn about which types of humor may be more beneficial than others. Newer publications suggested alternative scales that seem to be more sensible to assess differential effects of humor, such as the Humor Styles Questionnaire (HSQ) [136]. The HSQ includes several subscales with potentially more adaptive and maladaptive types of humor, as well as the newly developed Comic Style Markers [137], whose recorded scales offer new possibilities to shed further light into adaptive or maladaptive coping responses to heightened stress. Further research is needed to better understand the effects of different types of humor in the context of police officers coping with high levels of stress.

While the short Police Stress Questionnaire (PSQ) used herein provided for some clear results of an operational stress link with PTSD and organizational stress link with BO, the fact that a standalone study dedicated to the formal psychometric evaluation of said questionnaire is still needed, is a limitation. The initial reliability and validation results of the PSQ herein is comparable in quality to that of other short questionnaires like the TIPI [53,54,55]. Similar to the TIPI as a short version of the Big Five Personality Inventory [30,41], the PSQ herein can be viewed as an abridged version of longer versions of alternative questionnaires aimed to measure similar constructs [6,34,81], from which indeed the PSQ items herein were derived. In our cross-sectional study involving many scales and inventories, our abridged version of a PSQ was utilized in an effort to maintain appropriate response integrity from police officers in the study, through avoiding an excessively long questionnaire (which is also often why the TIPI may be utilized in such contexts of large sample, multifactorial study). Nonetheless, our assessment analyses on our abridged PSQ suggest that in future studies, further refinement of the scale, comparing it to full length alternatives [6,34,81] and considering other related job dimensions, would constitute an important research project in the field. For example, it is likely that operational and organisational stress are composed of subdimensions for which a short questionnaire is not well positioned to disentangle. It is therefore recommended that other studies, especially with fewer variables, utilize full-length alternatives of the PSQ and assess the relationships.

Two other criticisms can also be considered in respect to our study. In fact, they are also valid for all other studies carried out in the field. First, cross-sectional studies do not reveal the causal relationship between factors. We therefore cannot conclude that coping strategies remain strictly functional or dysfunctional since these strategies could arise in response to a pathology and not be the cause. For example, police officers with higher psychological trauma levels may be less likely to use humor than those without PTSD. Only a longitudinal study or a cohort study can objectively establish a list of coping strategies that are protective or determinant in the development of a mental pathology. Secondly, written questionnaires are not a substitute for diagnosis: they only collect indications of possible pathologies. No study on the mental health of police officers has so far been carried out using the diagnoses made by health professionals. In this respect, it would be relevant to combine psychometric evaluations with interview data like the Clinically Administered PTSD Scale (CAPS; [138]).

## 5. Conclusions

Based on a large sample of police officers, this study demonstrated that personality factors and coping strategies can be strongly associated with the presence or degree of PTSD and BO disorders. Consequently, specific variables within these categories may be instrumentally used to identify police officers at risk of developing mental disorders, versus those who are resilient from them. The father of offender profiling, Alexandre Lacassagne wrote: “Societies get the criminals they deserve” (p. 364, [139]). We can likewise ask ourselves this question in respect to police officers. Do societies get the police officers they deserve? One is not born, but rather becomes a police officer. Accordingly, it is our societal responsibility to recruit, train and protect our police officers. Appropriate preparation, prior training, team spirit and solid, positive professional relationships would guarantee less severe post-traumatic reactions [140], leading to positive work environments and consequently fewer cases of BO [118]. PTSD [141] and BO [142,143] have been associated with increased aggressivity and abusive policing practices. Taken together, it therefore makes utmost sense to take care of our police officers, in order to not only ensure the best police forces and services are provided to the population, but also prevent the use of violence during officer duty. Although some psychological preparedness training is already mandatory in the initial formation program of Swiss police officers, it is probably not sufficient: it should be maintained also throughout a number of years following, combined with improved intervention screenings, in order to better guarantee the mental health of police officers and, consequently, a better service to the population.

## Figures and Tables

**Table 1:** Descriptive Statistics, Positivity Thresholds and Counts for IES-R PTSD, MBI Burnout, MBI Emotional Exhaustion, HADS Anxiety, HADS Depression Scales and Suicidal Ideation.

	IES-R PTSD	MBI Burnout	MBI Emotional Exhaustion	HADS Anxiety	HADS Depression	BDI-II Suicidal Ideation
Mean (SD)	12.0 (13.4)	Multi-scale	12.9 (9.0)	5.6 (3.3)	3.2 (3.0)	0.14 (0.37)
IQR	2–17	-	17–50	3–7	1–5	0–0
Max	76	-	50	21	18	3
Threshold≥	24|33	Multi-scale	18	8	8	1
% Positive	16|9	16	25	25	9	14

Note: IES-R = Impact of Event Scale–Revised, MBI = Maslach Burnout Inventory, HADS = Hospital Anxiety and Depression Scale, BDI-II = Beck Depression Inventory; see Section 2 for a detailed explanation.

**Table 2:** Two-sample *t*-tests (presence vs. absence) for pathologies PTSD, Burnout, Suicidal Ideation, Anxiety and Depression (test statistic values and * *p* < 0.05; ** *p* < 0.01; *** *p* < 0.001 after Holm-Bonferroni correction). Above the first horizontal line, the pathological group had weaker values for these variables.

	IES-R PTSD	MBI Burnout	HADS Anxiety	HADS Depression	BDI-II Suicidal Ideation
% Positive	9%	16%	25%	9%	14%
BCope Reinterpretation Positive	−3.04 *	−7.34 ***	−7.44 ***	−6.57 ***	−4.63 ***
B5 Agreableness	−2.57	−7.76 ***	−4.52 ***	−4.37 ***	−4.17 ***
BCope Humour	−3.68 **	−3.08 *	−6.18 ***	−4.88 ***	−1.25
B5 Conscientiousness	−1.10	−4.88 ***	−3.05 *	−3.26 *	−1.49
B5 Extraversion	−0.85	−3.86 **	−2.43	−4.38 ***	−1.72
BCope Acceptance	−0.56	−4.82 ***	−3.57 **	−2.28	−1.57
BCope Active Coping	0.88	−4.21 ***	−0.70	−3.36 *	0.08
BCope Emotional Expression	1.09	−2.30	0.29	−3.00 *	−0.46
B5 Neuroticism	6.74 ***	11.11 ***	19.66 ***	8.30 ***	7.34 ***
PSQ Stress Organisational	4.98 ***	13.62 ***	10.09 ***	8.58 ***	5.14 ***
PSQ Life Imbalance	6.22 ***	8.72 ***	6.63 ***	8.67 ***	4.08 **
BCope Behavior Withdrawal	6.66 ***	7.08 ***	7.96 ***	7.07 ***	5.03 ***
BCope Blame	5.51 ***	5.23 ***	8.55 ***	6.50 ***	6.56 ***
BCope Substance Use	4.08 **	5.60 ***	6.14 ***	4.12 **	4.40 ***
BCope Denial	6.09 ***	3.93 **	5.70 ***	3.88 **	4.44 ***
PSQ Stress Operational	5.36 ***	5.94 ***	4.60 ***	4.16* *	2.24
BCope Distraction	7.91 ***	1.93	4.25 ***	0.07	1.89
BCope Religion	3.78 **	1.28	3.82 **	1.39	4.17 ***
Years of Service	1.59	0.79	2.57	2.50	6.24 ***
Age	1.82	0.48	1.98	1.39	5.36 ***
BCope Emotional Support	4.23 ***	−0.74	3.18 *	−1.02	0.76
Gender	0.11	0.33	−3.15 *	1.07	5.05 ***

Note: BCope = The Coping Orientation to Problems Experienced Inventory, B5 = Big Five Inventory in French, IES-R = Impact of Event Scale–Revised, MBI = Maslach Burnout Inventory, HADS = Hospital Anxiety and Depression Scale, BDI-II = Beck Depression Inventory, PSQ = Police Stress Questionnaire; see Section 2 for a detailed explanation.

**Table 3:** Linear multiple regression results for the prediction of total IES-R PTSD scores.

Variable	*B*	CI	*t*	*p*
B5 Openness	−0.12	[−0.17, −0.07]	−4.9	<0.001
BCope Humour	−0.09	[−0.14, −0.04]	−3.36	0.001
BCope Active Coping	−0.08	[−0.15, −0.01]	−2.33	0.02
PSQ Stress Operational	0.17	[0.12, 0.22]	6.38	<0.001
BCope Substance Use	0.14	[0.09, 0.19]	5.74	<0.001
BCope Behavior Withdrawal	0.14	[0.09, 0.19]	5.32	<0.001
BCope Distraction	0.14	[0.09, 0.19]	5.23	<0.001
BCope Religion	0.11	[0.06, 0.16]	4.47	<0.001
BCope Emotional Support	0.17	[0.10, 0.25]	4.44	<0.001
B5 Neuroticism	0.12	[0.07, 0.18]	4.38	<0.001
BCope Denial	0.10	[0.05, 0.15]	3.9	<0.001
BCope Emotional Expression	0.12	[0.05, 0.18]	3.52	<0.001
BCope Acceptance	0.08	[0.02, 0.13]	2.85	0.004

Note: BCope = The Coping Orientation to Problems Experienced Inventory, B5 = Big Five Inventory in French, IES-R = Impact of PSQ = Police Stress Questionnaire; see Section 2 for a detailed explanation.

**Table 4:** Logistic regression results for the prediction of BO pathology presence (as determined by the MBI subscales).

Variable	*B*	CI	*t*	*p*
BCope Active Coping	−0.88	[−1.31, −0.45]	−4.03	<0.001
B5 Agreableness	−0.51	[−0.79, −0.24]	−3.72	<0.001
B5 Conscientiousness	−0.48	[−0.76, −0.2]	−3.4	0.001
Bcope Reinterpretation Positive	−0.6	[−1.0, −0.2]	−2.96	0.003
Bcope Acceptance	−0.38	[−0.69, −0.08]	−2.44	0.02
PSQ Stress Organizational	2.15	[1.74, 2.55]	10.3	<0.001
B5 Neuroticism	1.55	[1.2, 1.91]	8.57	<0.001
PSQ Life Imbalance	0.82	[0.5, 1.13]	5.08	<0.001
Bcope Behavior Withdrawal	0.46	[0.23, 0.68]	3.9	<0.001
Bcope Distraction	0.42	[0.07, 0.77]	2.36	0.02
Bcope Substance Use	0.23	[0.0, 0.46]	2	0.04

Note: Bcope = The Coping Orientation to Problems Experienced Inventory, B5 = Big Five Inventory in French, MBI = Maslach Burnout Inventory, PSQ = Police Stress Questionnaire; see Section 2 for a detailed explanation.

## Data Availability

The data presented in this study are available on request from the corresponding authors.

## Supplementary Materials

The following supporting information can be downloaded at: https://www.mdpi.com/article/10.3390/ijerph19159218/s1, Table S1. Summary table and guide for all variables, Table S2. Two sample *t*-tests between clusters for all variables in Figure 3, Table S3. Short version Police Stress Questionnaire (PSQ) items.
