# Supplementary material for: Profiling Police Forces against Stress: Risk and Protective Factors for Post-Traumatic Stress Disorder and Burnout in Police Officers
Source: Int J Environ Res Public Health. 2022 Jul 28;19(15):9218. doi: 10.3390/ijerph19159218 (PMC9368562; doi:10.3390/ijerph19159218)
Supplement: Supplementary file 1 [file ijerph-19-09218-s001.zip › ijerph-1789314-supplementary.pdf]

Supplementary Materials
Profiling Police Forces against Stress: Risk and Protective Factors for Post-Traumatic Stress Disorder and Burnout in Police Officers

Table S1. Summary table and guide for all variables

Variable	Measure or Interpretation	Coding	*M (SD)* or Freq
**BCope Acceptance**	Acceptance coping strategy	2 to 8	6.0 (1.7)
**BCope Blame**	Blame coping strategy	2 to 8	3.6 (1.4)
**BCope Active**	Active coping strategy	2 to 8	4.8 (1.7)
**BCope Denial**	Denial coping strategy	2 to 8	2.3 (0.8)
**BCope BehaviorWithdrawal**	Behavioral withdrawal coping strategy	2 to 8	2.5 (0.9)
**BCope Distraction**	Distraction coping strategy	2 to 8	4.4 (1.6)
**BCope EmotionExpression**	Emotional expression coping strategy	2 to 8	4.7 (1.5)
**BCope Humor**	Humor coping strategy	2 to 8	3.9 (1.5)
**BCope Planning**	Planning coping strategy	2 to 8	4.4 (1.7)
**BCope Religio**	Religion coping strategy	2 to 8	2.8 (1.5)
**BCope ReinterpPositive**	Positive reinterpretation coping strategy	2 to 8	5.4 (1.7)
**BCope EmotionalSupport**	Emotional support coping strategy	2 to 8	4.1 (1.6)
**BCope InstrumentalSupport**	Instrumental support coping strategy	2 to 8	4.2 (1.6)
**BCope Substance Use**	Drugs or substance use coping strategy	2 to 8	2.5 (1.0)
**B5 Agreeableness**	Big-5 Agreability trait	5 to 50	41.7 (4.8)
**B5 Conscientiousness**	Big-5 Conscientiousness trait	5 to 50	38.5 (4.5)
**B5 Extraversion**	Big-5 Emotional Stability trait	5 to 50	28.0 (6.2)
**B5 Neuroticism**	Big-5 Extraversion trait	5 to 50	17.5 (6.0)
**B5 Openness**	Big-5 Openness trait	5 to 50	35.6 (6.0)
**HADS Anxiety**	Degree of Anxiety	0 to 21	5.6 (3.3)
**HADS Depression**	Degree to Depression	0 to 21	3.2 (3.0)
**BDI-II Suicidal Ideation**	Degree of suicidal consideration	0 to 3	0.1 (0.4)
**IES-R Avoidance**	PTSD avoidance subscale	0 to 32	4.1 (5.1)
**IES-R Hyperarousal**	PTSD hyperarousal subscale	0 to 24	1.9 (3.4)
**IES-R Intrusion**	PTSD intrusion subscale	0 to 32	5.9 (6.3)
**IES-R PTSD**	PTSD total score	0 to 88	12.0 (13.4)
**MBI Accomplishment**	Degree of accomplishment at work	0 to 48	33.8 (8.6)
**MBI Depersonalisation**	Degree of depersonalization at work	0 to 30	9.4 (6.5)
**MBI Emotional Exhaustion**	Degree of exhaustion at work	0 to 54	12.9 (9.0)
**PSQ StressOperational**	Degree of operational stress for police work	5 to 25	17.9 (3.6)
**PSQ StressOrganisational**	Degree of organizational stress for police work	5 to 25	16.0 (3.6)
**PSQ LifeImbalance**	Degree of life imbalance	1 to 5	3.3 (1.2)
**Age**	Age in years	22 to 65	39.9 (9.4)
**Gender**	Binary, Female, Male (coded 1)	0 ǀ 1	78% ǀ 22%
**Years of Service**	Number of years employed as an officer	0 to 43	15.6 (10.1)
**Relational Status**	Binary, Single, In a Couple (coded 1)	0 ǀ 1	78% ǀ 22%
**Num Children**	Ordinal, Number of children	0 to >3	0.9 (1.1)

Table S2. Two sample t-tests between clusters for all variables in Figure 3.

Variable Clusters :	1 - 2	1 - 3	2 - 3
**BCope ReinterpPositive**	12.32***	11.77***	5.78***
**MBI Accomplishment**	8.66***	3.38*	-0.30
**B5 Agreeableness**	8.11***	0.46	-4.00**
**BCope Acceptance**	7.08***	12.60***	8.83***
**B5 Conscientiousness**	6.64***	0.59	-2.71
**BCope Humor**	6.43***	7.38***	3.98**
**B5 Extraversion**	5.42***	0.45	-2.29
**BCope Active**	4.60***	24.29***	18.50***
**B5 Openness**	3.57**	0.68	-0.96
**BCope Planning**	3.25*	27.90***	21.51***
**IES-R Hyperarousal**	-23.84***	10.39***	31.85***
**MBI Emotional Exhaustion**	-20.60***	3.34*	13.42***
**IES-R Avoidance**	-20.47***	10.74***	25.85***
**IES-R Intrusion**	-20.41***	7.51***	20.84***
**HADS Depression**	-19.96***	1.32	11.04***
**HADS Anxiety**	-18.88***	5.64***	15.48***
**B5 Neuroticism**	-15.09***	2.48	9.42***
**PSQ Stress Organisational**	-14.58***	2.71	10.18***
**MBI Depersonalisation**	-12.34***	2.09	9.17***
**BCope Behavior Withdrawal**	-12.15***	1.70	9.71***
**PSQ LifeImbalance**	-11.95***	1.63	7.49***
**BCope Substance Use**	-10.61***	4.55***	12.67***
**PSQ Stress Operational**	-10.06***	2.66	7.26***
**BDI-II Suicidal Ideation**	-8.41***	1.90	8.44***
**BCope Denial**	-7.82***	3.20*	8.90***
**BCope Blame**	-7.82***	14.24***	18.84***
**BCope Distraction**	-4.74***	16.97***	19.87***
**BCope Religion**	-2.79*	7.52***	8.78***
**BCope Emotion Expression**	2.43	18.54***	15.59***
**Num Children**	-2.36	1.75	2.93*
**Years of Service**	-1.93	-0.83	0.04
**BCope Emotion Support**	-1.68	25.72***	22.99***
**Age**	-1.31	-0.97	-0.32
**BCope InstrumentalSupport**	1.18	21.74***	17.59***
**Relational Status**	-0.58	0.48	0.74
**Gender**	-0.10	-1.54	-1.40

Table S3. Short version Police Stress Questionnaire (PSQ) items

Items
Operational Stress Subscale
1. My job takes a lot out of me emotionally<br>*Mon travail me demande beaucoup émotionnellement*
2. My job takes a lot out of me mentally and intellectually<br>*Mon travail me demande beaucoup mentalement et intellectuellement*
3. I regularly face physical threats<br>*Je suis régulièrement confronté à des menaces physiques*
4. I regularly respond to emergency situations<br>*J'ai régulièrement des interventions urgentes*
5. I feel a lack of public gratitude<br>*Je ressens un manque d'estime du publique*
Organisational Stress Subscale
6. I feel a lack of support from my superiors and my organization<br>*Je ressens un manque de soutien de la part de mes supérieurs et de mon organisation*
7. I feel a lack of support from my colleagues<br>*Je ressens un manque de soutien de la part de mes collègues*
8. I consider my workload to be excessive<br>*Selon moi, la charge de travail est excessive*
9. I feel that our organization is understaffed<br>*Selon moi, notre organisation est en manque d'effectifs*
10. I am not paid enough for the work I do<br>*Je ne suis pas assez payé-e par rapport au travail que je fais*
Life Balance
11. Balancing my family life and professional life is difficult<br>*Concilier ma vie de famille et ma vie professionnelle est difficile*

Note: All questions asked on a Likert scale 1 to 5 ("Strongly Disagree to Strongly Agree")
